# The Correlation between Oral Health and Air Pollution: A Systematic Review

**DOI:** 10.3390/dj12070215

**Published:** 2024-07-11

**Authors:** Bruna Sinjari, Manlio Santilli, Piero Di Carlo, Eleonora Aruffo, Sergio Caputi

**Affiliations:** 1 Unit of Prostodontics, Department of Innovative Technologies in Medicine and Dentistry, University “G. d’Annunzio” Chieti-Pescara, 66100 Chieti, Italy; manlio.santilli@unich.it (M.S.); scaputi@unich.it (S.C.); 2 Center of Advanced Studies and Technology (CAST), University of “G. d’Annunzio” Chieti-Pescara, 66100 Chieti, Italy; piero.dicarlo@unich.it (P.D.C.); eleonora.aruffo@unich.it (E.A.)

**Keywords:** oral health, air pollution, periodontitis, dentistry, systematic review

## Abstract

This systematic review assessed to evaluate the potential correlation between oral health and air pollution. To the best of the authors’ knowledge, this is the first systematic review endeavoring to compare air pollution and oral health. A systematic search was performed according to the PRISMA (Preferred Reporting Items for Systematic Reviews and Meta-analyses) statement and employed the PICO(S) approach (Patient or Population, Intervention, Control or Comparison, Outcome, and Study types). The search was limited to English-language articles, and publications within a 15-year timeframe were included in the electronic search. A comprehensive search was conducted across PubMed, Scopus, Embase, and Web of Science databases, spanning the years 2008 to 2023, resulting in a total of 4983 scientific articles. A final selection of 11 scientific papers was made based on their study type and the specific air pollutants examined. The selected papers analyzed various air pollutants associated with health-related diseases, including Ozone, Nitrogen Dioxide, Nitrogen Monoxide, Carbon Monoxide, sulfur dioxide, and particulate matter. Three out of eleven of the reviewed studies assert a strong correlation between air pollutants and oral diseases, specifically periodontitis. However, the exact biological mechanisms underlying this correlation do not seem to be fully understood, indicating the need for further comprehensive investigation in this regard. Dentists can contribute to the collective effort by educating their patients about the oral health implications of air pollution, thereby supporting initiatives aimed at promoting environmental and health sustainability.

## 1. Introduction

Environmental pollution refers to the release or production of harmful substances or contaminants into the natural environment, such as air, water, or soil, which can have detrimental effects on living organisms and ecosystems. Air pollution, specifically, is a complex mixture of particles and gases that can pose significant risks to human health [1]. It arises from various sources, including industrial emissions, transportation activities, and household practices. The World Health Organization (WHO) estimates that air pollution contributes to approximately 7 million premature deaths worldwide annually [2]. Unfortunately, the European Environment Agency shares this concerning perspective, highlighting the substantial impact of air pollution on the health of the population, particularly in urban areas [3]. Despite notable progress in reducing the emission of primary air pollutants and improving air quality over the past two decades in Europe, thanks in part to the collective efforts toward achieving the 17 Sustainable Development Goals (SDGs) by 2030 [4], many regions still face inadequate air quality. In the previous year, air pollution led to a significant number of premature deaths across the 27 EU Member States (EU-27). Exceeding the World Health Organization’s guideline for the inhalation of fine particulate matter with an aerodynamic diameter of ≤2.5 μm (PM_2.5_) resulted in 238,000 premature deaths, and exposure to nitrogen dioxide (NO_2_) levels beyond the recommended limit led to 49,000 premature deaths. Additionally, acute exposure to ozone caused 24,000 premature deaths [2,3]. The gravity of the issue is further emphasized when considering the morbidity effects [2,3].

The WHO has identified PM, NO_2_, sulfur dioxide (SO_2_), and ground-level ozone (O_3_) as the air pollutants with the greatest potential to harm human health [2]. These pollutants have been linked to various health complications, including respiratory and cardiovascular diseases, negative birth outcomes, and neurological disorders [1,5,6]. PM, in particular, is considered highly hazardous in air pollution due to its ability to penetrate deep into the lungs, causing inflammation and tissue damage [1]. Nitrogen oxides (NO_x_) and SO_2_, among other pollutants, also have detrimental effects on human health [1,7], contributing to respiratory issues, exacerbating cardiovascular diseases, and even increasing the risk of premature mortality. Moreover, long-term exposure to air pollution has been associated with the development of chronic health conditions such as asthma, lung cancer, and heart disease [1,5,7,8]. These chronic illnesses further highlight the serious implications of sustained exposure to air pollutants. The impact of air pollution on health is particularly significant in vulnerable populations, including children, the elderly, and individuals with pre-existing health conditions [8]. Given the substantial effects of air pollution on health, there is a growing imperative for effective policies and interventions aimed at reducing pollution levels and safeguarding public health. Numerous studies have been conducted over the years to investigate the impact of air pollution on health, focusing on the associated risks [5,7,8,9].

However, there is a noticeable lack of research on the correlation between air pollution and oral health-related pathologies. This represents an intriguing finding that warrants further exploration, considering that inhalation and ingestion are the primary routes of exposure to these air pollutants. It is therefore reasonable to assume a close connection between these pollutants and the oral cavity. However, there is currently a dearth of studies, and notably, no systematic review has been conducted on this specific topic. In this context, dentists can contribute to the collective effort by educating their patients about the oral health implications of air pollution, thereby supporting initiatives aimed at promoting environmental and health sustainability. Consequently, the aim of this literature review is to examine the potential relationship between air pollution and oral health. To the best of the authors’ knowledge, this is the first systematic review endeavoring to compare air pollution and oral health.

## 2. Materials and Methods

### 2.1. Study Characteristics

**Figure 1:**
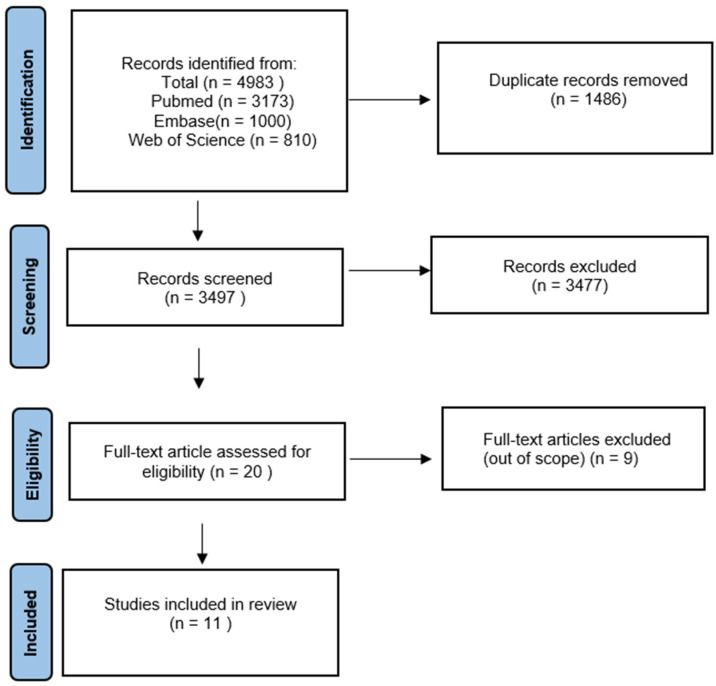
PRISMA flow diagram.

The article selection process for this review followed the guidelines provided by the PRISMA flow diagram, as shown in Figure 1. A comprehensive search was conducted across PubMed, Scopus, Embase, and Web of Science databases, spanning 1 January 2008 to 31 December 2023 (a 15-year timeframe was included), resulting in a total of 4983 scientific articles. To eliminate duplicates, the references of the identified records were uploaded to the digital tool Rayyan (http://rayyan.qcri.org, accessed on 23 May 2023). The systematic review is registered in the International Prospective Register of Systematic Reviews, Prospero, under the identification CRD 42024559844. After duplicate removal, 3477 unique papers remained for further analysis. The titles of these articles were manually reviewed to include relevant references related to the correlation between air pollutants and oral health. Following this screening process, 20 studies were identified as relevant to the topic of air pollutants and their impact on oral health. After that, 9 studies were excluded (did not meet our inclusion criteria); a final selection of 11 scientific papers was made based on their study type and the specific air pollutants examined.

### 2.2. Search Strategy

This systematic review adhered to the guidelines outlined in the PRISMA (Preferred Reporting Items for Systematic Reviews and Meta-analyses) statement and employed the PICO(S) approach (Patient or Population, Intervention, Control or Comparison, Outcome, and Study types). Extensive literature research was conducted using the databases PubMed (Medline), Embase, Web of Knowledge, and Cochrane Library, focusing on the correlation between air pollution and oral health. The search strategy utilized various combinations of keywords, including “Air pollution” OR “environmental” OR “pollution” AND “dental health” OR “oral health”. The PICO question was formulated as follows: P-population: individuals exposed to air pollution; I-intervention: measures undertaken by dental practitioners to comprehend the relationship between air pollution and oral health; C-comparison: no intervention or standard care; and O-outcome: oral health outcomes associated with air pollution such as dental caries, periodontitis, and related conditions. This question specifically investigates the association between air pollution and oral health, as well as the role of dental practitioners in addressing this issue. The search was limited to English-language articles, and publications within a 15-year timeframe were included in the electronic search.

The research questions of this systematic review were:

How many studies have been published on the effects of air pollution on oral health?
What methodological characteristics did the paper publish?
Which air pollutants were commonly investigated?
Is it possible to consider a relation between oral health outcomes and air pollutants?

### 2.3. Inclusion and Exclusion Criteria

Articles were considered appropriate when satisfying the following inclusion criteria:

1. Articles with main topic regarding the correlation between air pollution and their impact on oral health;
2. Studies performed in vivo;
3. Full-text paper in English;
4. Retrospective study, case-control study, cross-sectional study were included.

Articles that did not have the above information were excluded from the review. Research letters and conference proceedings were excluded. Air pollutant exposure of workers in dental clinic/hospital were excluded.

### 2.4. Data Extraction and Analysis of the Data

All the relevant data were extracted, including the author’s name, publication year, type of study, pollutants measured, and country and impact of oral health. Specifically, a data abstraction sheet was used, and the accuracy of the information was verified by both B.S. and M.S. (in accordance with all the authors). The investigated pollutants were classified as follows:

Gaseous/air pollutants (such as O_3_ = Ozone; NO = Nitrogen Monoxide; NO_2_ = Nitrogen Dioxide; SO_2_ = Sulfur Dioxide; CO = Carbon Monoxide) and particulate matter (i.e., PM_10_, PM_2.5_).

## 3. Results

The 11 selected papers analyzed various air pollutants associated with health-related diseases, including Ozone, Nitrogen Dioxide, Nitrogen Monoxide, Carbon Monoxide, sulfur dioxide, and particulate matter. Notably, particulate matter with an aerodynamic diameter of ≤10 μm diameters (PM_10_) and PM_2.5_ were included in the analysis, as outlined in Table 1. The studies underwent quality assessment utilizing the QUADAS-2 tool, as outlined in Table 1. All publication were classified as having a high risk of bias (Table 2).

**How many studies have been published on the effects of air pollution on oral health?**

Upon conducting a systematic review, it was found that the initial study exploring the association between oral health and air pollution was published in 2008 in Taiwan. That study specifically investigated the correlation between oral cleft lip, either with or without palate and air pollution. Out of the total of eleven studies reviewed, a significant association between maternal exposure to air pollution and oral clefts was observed in seven studies. However, the primary focus of the authors was to primarily examine pathologies directly related to the oral cavity and within the domain of dental expertise. The first study establishing a relationship between periodontitis and air pollution was published in 2014. Among the eleven studies analyzed, three studies demonstrated an association between periodontitis and air pollution. This finding is summarized in Table 1, indicating an increasing over the past five years in publications suggesting a correlation between periodontitis and air pollution within the last decade. Only one out of the eleven studies reviewed addressed the correlation between oral cancer and air pollution.

**What methodological characteristics did the paper publish?**

The methodological characteristics are presented in Table 3.

### Geographical Distribution

**Figure 2:**
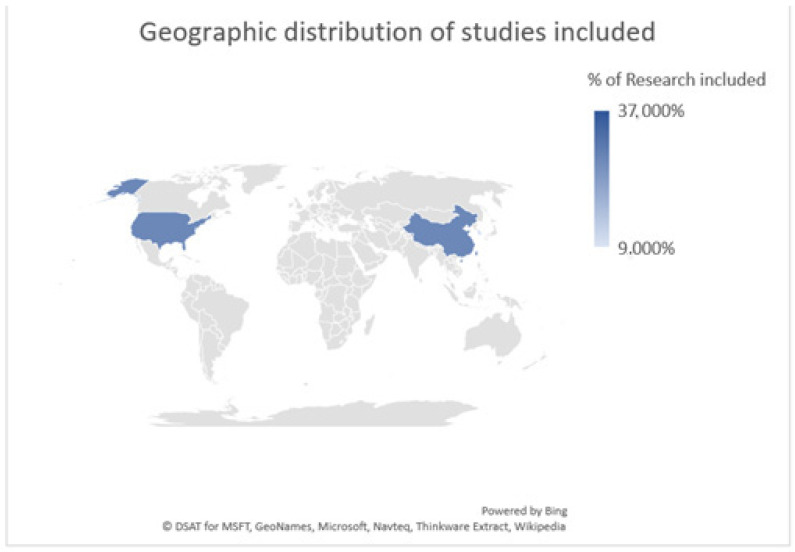
Geographic distribution of studies included: Taiwan (n = 4) 37%; United States (n = 3) 27%; China (n = 3) 27%; South Korea (n = 1) 9%.

Concerning the geographical distribution, data pertaining to the correlation between air pollution and oral health have been published by institutions in various countries across the globe. The studies included in this systematic review specifically originate from Taiwan (n = 4), the United States (n = 3), China (n = 3), and South Korea (n = 1), as illustrated in Figure 2.

**Which air pollutants were commonly investigated?**

The air pollutants investigated in the current study are presented in Table 4.

## 4. Discussion

**Is there a relationship between oral health outcomes and air pollutants?**

This systematic review is the first to examine the association between common air pollutants (CO, O_3_, NO, SO_2_, and PM) and oral health, highlighting that these pollutants primarily enter the body through inhalation, causing various respiratory and cardiovascular problems [30,31,32,33,34,35,36,37,38,39,40,41,42,43,44]. Notably, particulate matter (PM) can deeply penetrate the lungs and bloodstream, leading to severe health issues like premature death, non-fatal heart attacks, and aggravated asthma [45,46,47,48,49]. The available literature consistently demonstrates that inhalation and ingestion are the primary routes of exposure to air pollutants [46,47,48,49]. As a result, it is reasonable to assume a strong correlation between these pollutants and their impact on the oral cavity. Nevertheless, it is important to note that there is a scarcity of studies specifically focusing on this association, hence the idea to investigate this possible relationships. Three out of eleven studies selected in this review support the existence of an association between oral health and air pollution. In the last 15 years, there was an increase in publications that support this assumption. Specifically, the first study was published in 2014 by Tsung-Han Yang et al., who investigated via a cross-sectional study the association between particulate matter (PM_2.5_) and high-sensitivity C-reactive protein (hs-CRP), assuming that patients with periodontal diseases had higher CRP levels compared to periodontally healthy subjects. The researchers reached the conclusion that this correlation is feasible since both periodontal diseases and particulate air pollution have been associated with a higher likelihood of experiencing systemic inflammation and oxidative stress. Moreover, they share a common exposure pathway. 

Essentially, periodontal inflammation could potentially cause a systemic rise in inflammatory markers, which subsequently amplifies the body’s reaction to particulate air pollution in the lungs. Recently, in 2021, Han Jie-Lin et al. realized a retrospective cohort study using longitudinal generation tracking and the Taiwan air quality-monitoring databases, thus concluding that resident in Taiwan with long-term exposure to higher levels of air pollutants (SO_2_, CO, NO, NO_2_, NO_x_, PM_2.5_, and PM_10_) had a greater risk of periodontitis. The authors, however, arrived at a conclusion very similar to that of their predecessors, namely, that the precise mechanisms establishing a connection between periodontitis and air pollution are not yet fully understood, despite extensive research on various air pollutants and their associations with numerous systemic effects. These effects include, among others, increased systemic oxidative stress, increased blood coagulation, and decreased plasma antioxidant capacity [11,50].

In a recently published paper in 2023, Marruganti et al. aimed to further investigate the epidemiological connection between periodontitis and air pollution [10]. They conducted a cross-sectional study in a representative sample of population of South Korea, where they analyzed the average annual levels of PM_10_, O_3_, SO_2_, and NO_2_. Their findings revealed that even a slight increase in outdoor levels of PM_10_ and O_3_ were positively associated with prevalence of periodontitis [10]. On the other hand, NO_2_ and humidity showed an inverse correlation with periodontitis. The results remained consistent when considering severe cases of periodontitis, showing a similar association with SO_2_ and NO_2_. The authors discussed three potential mechanisms that could be associated with periodontitis: inflammation, oxidative stress, and alterations in the microbiome. Specifically, according to this study, the accumulation of particles and components of air pollutants (such iron, copper, and zinc) on the oral mucosa and periodontium can potentially activate comparable inflammatory pathways and induce oxidative stress within the oral cavity [10]. This process mirrors the mechanisms observed in the lungs. At present, however, one fact emerges, namely that, although there is a correlation of some air pollutants, especially particulate matter, with periodontitis, the underlying pathophysiological mechanisms have not yet been explained and clarified. Thus, much more research should be conducted in this regard.

Another finding important to analyze is the correlation between oral cleft and air pollutants, a finding that was common to as many as seven of the eleven articles analyzed, although the main objective of the present systematic review was to investigate the relationship of pollutants with oral diseases. Briefly, orofacial clefts are common congenital malformations generally subdivided into two types: cleft palate only (CPO) and cleft lip with or without cleft palate (CL/P). In a case-control study conducted by Jiang Wen et al. in 2021, it was hypothesized that maternal exposure to CO, NO_2_, and SO_2_ during the first trimester of pregnancy could potentially play a role in the occurrence of orofacial clefts. The researchers suggested that these associations might be independent from particulate matter, highlighting the specific focus on these gaseous pollutants in relation to the development of orofacial clefts [12]. In contrast, Liu-fang-Hua’s research in 2020 presented evidence supporting the notion that exposure to PM_10_ during the three months prior to conception and the first trimester of pregnancy raises the likelihood of developing oral clefts [13]. Several other authors, including Jinzhou Zhao in China and Ying Zhou in the United States, have reported similar associations between PM_2.5_, PM_10_, O_3_, CO, and SO_2_ exposure and the risk of oral clefts [14]. These studies have shown that PM_2.5_ significantly increases the risk of cleft palate alone, but does not affect the incidence of cleft lip with or without palate [14]. Additionally, research conducted by Marshall et al. and Tanner in the United States and other studies have found limited and inconsistent evidence linking maternal exposure to ambient air pollutants with cleft malformations [17,19]. However, the majority of the authors reached the consensus that the biological mechanisms responsible for the connections between gaseous air pollutants and orofacial clefts still remain unclear. Some proposed mechanisms include: (1) mutations in fetal DNA that disrupt cellular apoptosis, (2) instances of oxygen deprivation (anoxic events), (3) oxidative damage, and (4) toxicity targeting specific fetal cell populations [51]. In one study out of the eleven, the association between PM_2.5_ and oral cancer was examined. The authors suggest that elevated levels of PM_2.5_ might be linked to a higher risk of oral cancer [15]. However, the exact mechanism by which this relationship exists is not fully comprehended, although for some authors the carcinogenicity of PM_2.5_ has been linked to oxidative DNA damage, metabolism of organic compounds, and inflammatory lesions [52].

### Study Limitations

This study has several limitations. Firstly, the exact concentrations of PM_10_, PM_2.5_, and other pollutants analyzed, such as CO, SO_2_, NO_2_, and O_3_, are not well specified in every study. As a result, interpreting all the data with absolute clarity becomes challenging. In several selected studies, the levels of air pollutants were assessed using a database that gathers data from air monitoring stations located across the region. In addition, the variations in findings among studies could be attributed, at least in part, to disparities in the levels and ranges of air pollutants. Nevertheless, a substantial portion of the reviewed studies assert a strong correlation between air pollutants and oral diseases, specifically periodontitis. However, the exact biological mechanisms underlying this correlation do not seem to be fully understood, indicating the need for further comprehensive investigation in this regard.

## 5. Conclusions

In conclusion, this systematic review highlights a potential link between air pollution and oral health, specifically pointing to a significant correlation with periodontitis as evidenced in some studies. However, the limited understanding of the underlying biological mechanisms underscores the necessity for further research to elucidate these connections. By raising awareness and educating patients about the detrimental effects of air pollution on oral health, dentists can help advance public health initiatives.

## Figures and Tables

**Table 1:** Table summarizing the principal results of the study. PM = Particulate Matter; O_3_ = Ozone; NO = Nitrogen Monoxide; NO_2_ = Nitrogen Dioxide; SO_2_ = Sulfur Dioxide; CO = Carbon Monoxide.

Author	Year	Type of Study	Pollutant	PM (Particulate Matter)	Country	Impact on Oral Health	Risk of Bias
Marruganti et al. [10]	2023	Cross-sectional study	O_3_ NO_2_ SO_2_	PM_<10_	South Korea	Periodontitis	High
Han-Jie Lin et al. [11]	2021	Retrospective cohort study	NO NO_2_ SO_2_	PM_2.5_ PM_10_	Taiwan	Periodontitis	High
Jiang Wen [12]	2021	Case-control study	CO O_3_ NO_2_ SO_2_	PM_2.5_ PM_10_	China	Oral Cleft	High
Liu Fang-Hua [13]	2020	Population-based, case-control study	N.A.	PM_10_	China	Oral cleft in offspring	High
Jinzhu Zhao [14]	2018	Prospective population-based cohort study	O_3_ SO_2_ CO	PM_2.5_ PM_10_	China	Oral Cleft	High
Yu-Hua Chu et al. [15]	2018	Retrospective study	O_3_ NO_2_ SO_2_ CO NO	PM_10_ PM_2.5_ PM_10–2.5_	Taiwan	PM_2.5_ and oral cancer	High
Ying Zhou [16]	2017	Retrospective study	O_3_	PM_2.5_	USA	Oral cleft	High
Tanner Jean Paul [17]	2015	Retrospective cohort study	Benzene	PM_2.5_	USA	Oral Cleft	High
Tsung-Han Yang [18]	2014	Cross-sectional study	N.A.	PM_2.5_	Taiwan	Periodontitis	High
Marshall Elizabeth G. [19]	2009	Case-control study	O_3_ CO NO_2_	PM_10_ PM_2.5_	USA	Oral cleft malformations	High
Hwang Bing-Fang [20]	2008	Population-based case-control study	SO_2_ NO_x_ O_3_	PM_10_	Taiwan	Cleft Lip with or without palate	High

**Table 2:** QUADAS-2 tool quality assessment, ** high risk of BIAS.

Author	Patient Selection	Index Test	Reference Standard	Flow and Timing
Marruganti et al. [10]	**	**	**	**
Han-Jie Lin et al. [11]	**	**	**	**
Jiang Wen [12]	**	**	**	**
Liu Fang-Hua [13]	**	**	**	**
Jinzhu Zhao [14]	**	**	**	**
Yu-Hua Chu et al. [15]	**	**	**	**
Ying Zhou [16]	**	**	**	**
Tanner Jean Paul [17]	**	**	**	**
Tsung-Han Yang [18]	**	**	**	**
Marshall Elizabeth G. [19]	**	**	**	**
Hwang Bing-Fang [20]	**	**	**	**

**Table 3:** Table summarizing the principal results of the study. N.A. = Not applicable; OR = Odds ratio.

Author	Sample Size	Study Design	Pollutant and PM	Age (Years) and Gender, N(%)	Resume of Principal Results
Marruganti et al. [10]	42,020	Cross-sectional survey	O_3_ NO_2_ SO_2_ PM_<10_	44.91 M 17,876 (49.6%) W 24,144 50.4%)	An increase of 5 µg/m^3^ in PM_10_ concentration was associated with a higher prevalence of periodontitis (OR = 1.17; 95% confidence interval—CI: 1.11–1.24). Similarly, a 5 ppb increase in O_3_ levels was also positively associated with periodontitis prevalence, with an odds ratio of 1.4 (95% CI: 1.00–1.30). On the other hand, a 5% increase in humidity showed an inverse association with periodontitis (OR = 0.94; 95% CI: 0.90–0.99), meaning that higher humidity levels were associated with a lower prevalence of periodontitis. Additionally, a 3 ppb increase in NO_2_ levels was inversely associated with periodontitis, with an odds ratio of 0.93 (95% CI: 0.89–0.96), indicating that higher NO_2_ levels were associated with a lower prevalence of periodontitis.
Han-Jie Lin et al. [11]	292,263	Retrospective cohort study	NO NO_2_ SO_2_ PM_2.5_ PM_10_	41.1 M 131,278 (44.9%) W 160,985 (55.1%)	The incidence of periodontitis increased with exposure to SO_2_, CO, NO, NO_2_, NO_x_, PM_2.5_, and PM_10_. The concentrations of these air pollutants were divided into quartiles (Q1, Q2, Q3, and Q4). After adjusting for age, sex, population density, and comorbidities, the adjusted hazard ratios (95% confidence interval—CI%) for periodontitis in Q2–Q4 showed a significant increase with higher levels of exposure to SO_2_, CO, NO, NO_2_, NO_x_, PM_2.5_, and PM_10_, respectively.
Jiang Wen [12]	446 Case 4460 Control	Case-control study	CO O_3_ NO_2_ SO_2_ PM_2.5_ PM_10_	N.A. Case M 272 W 174 Control M 2325 W 2135	An increase in CO, NO_2_, and SO_2_ exposure significantly elevated the risk of cleft lip with or without cleft palate (CL/P) in all months of the first trimester of pregnancy, with odds ratios ranging from 1.39 to 1.48 for CO, 1.35 to 1.61 for NO_2_, and 1.22 to 1.35 for SO_2_. The risk of cleft palate only (CPO) also increased with higher levels of NO_2_ exposure during the first trimester, with odds ratios ranging from 1.60 to 1.66. However, no significant effect of O_3_ exposure was observed in relation to the risk of oral clefts.
Liu Fang-Hua [13]	3086 Case 7950 Control	Population-based, case-control study	N.A. PM_10_	N.A. Case M 1743 W 1343 Control M 4023 W 3927	Maternal exposure to PM_10_ showed a positive association with an increased risk of oral cleft during the three months before conception (per 10 μg/m^3^ increase: OR = 1.04, 95% CI 1.01 to 1.07; highest quartile vs. lowest quartile: OR = 1.23, 95% CI 1.04 to 1.45) and the first trimester of pregnancy (per 10 μg/m^3^ increase: OR = 1.05, 95% CI 1.02 to 1.08; highest quartile vs. lowest quartile: OR = 1.37, 95% CI 1.15 to 1.64). Similar positive associations were observed in the analysis of individual months, with the highest quartile versus lowest quartile showing a particularly strong association in the second month of pregnancy (OR = 1.77, 95% CI 1.51 to 2.09).
Jinzhu Zhao [14]	105,927	Prospective population-based cohort study	O_3_ SO_2_ CO PM_2.5_ PM_10_	N.A.	The aim of this study was to assess whether increased levels of maternal exposure to PM_2.5_, PM_10_, O_3_, CO, and SO_2_ are linked to a higher risk of oral clefts in Wuhan, China. The results revealed significant associations between PM_2.5_ exposure and both outcomes (CPO and CLP), particularly during the second month of pregnancy (aORs = 1.29 per 10 μg/m^3^ change; 95% CI: 1.17–1.42), as well as the third month of pregnancy (aORs = 1.11; 95% CI: 1.01–1.22). Moreover, the risk of having a baby with an oral cleft increased by 11–29% within the range of PM_2.5_ concentrations considered in the study. Similarly, significant associations were observed between CLP and exposure to O_3_ and PM_10_. Additionally, a significant association was found between CPO and exposure to CO.
Yu-Hua Chu et al. [15]	482,559	Retrospective study	O_3_ NO_2_ SO_2_ CO NO PM_10_ PM_2.5_ PM_10–2.5_	N.A.	The researchers assessed the levels of CO, O_3_, CO, and SO_2_, NO_x_ (PM_10–2.5_), and PM_2.5_ in 2009, categorizing them into quartiles. The analysis included a total of 482,659 men aged 40 years and above. Logistic regression was employed to examine the association between PM_2.5_ and oral cancer cases diagnosed between 2012 and 2013. After adjusting for factors such as PM_10–2.5_, SO_2_, O_3_, age, and betel quid chewing, the odds ratios (ORs) for oral cancer were found to be 0.91 (95% CI 0.75 to 1.10) for PM_2.5_ levels between 26.74 and 32.37 μg/m^3^, 1.00 (95% CI 0.84 to 1.20) for levels between 32.37 and 40.37 μg/m^3^, and 1.42 (95% CI 1.17 to 1.73) for levels ≥ 40.37 μg/m^3^. The association between PM_2.5_ and oral cancer risk remained unchanged even after further adjustment for smoking. In both models, PM_10–2.5_ and SO_2_ did not show a significant association with oral cancer, irrespective of their concentrations. However, O_3_, frequent betel quid chewing and occasional and frequent smoking were found to be significantly linked to oral cancer.
Ying Zhou [16]	4.7 Million	Retrospective study	O_3_ PM_2.5_	N.A.	The researchers found that for every 10 μg/m^3^ increase in PM_2.5_ concentration, there was a significant association with cleft palate alone (odds ratio, OR = 1.43, 95% confidence interval, CI: 1.11–1.86). However, they did not observe a significant association between PM_2.5_ concentration and cleft lip with or without cleft palate. Furthermore, no associations were found between ozone exposure and the two outcomes of orofacial clefts. The study suggests that increased levels of PM_2.5_ significantly increase the risk of cleft palate alone, but do not affect the incidence of cleft lip with or without palate.
Tanner Jean Paul [17]	2,123,874	Retrospective cohort study	Benzene PM_2.5_	N.A.	The findings revealed that mothers who had the highest level of benzene exposure had a higher likelihood of giving birth to an infant with an isolated cleft palate (adjusted prevalence ratio, 4th quartile: 1.52; 95% confidence interval, CI: 1.13–2.04) or any orofacial cleft (adjusted prevalence ratio, 4th quartile: 1.29; 95% CI: 1.08–1.56).
Tsung-Han Yang [18]	200	Cross-sectional study	N.A. PM_2.5_	M = 100 (50%) W = 100 (50%)	The authors discovered that for every 10 μg/m^3^ rise in the 24 h average of PM_2.5_, there was a 3.22% increase (95% confidence interval, CI: 1.21, 5.23; *p* < 0.01) in high-sensitivity C-reactive protein (hs-CRP) levels and a 1.03% increase (95% CI: 0.01, 2.05; *p* < 0.01) across all study participants. Among adult patients with chronic periodontitis, there was an 8.45% increase in hs-CRP and a 2.57% increase in the oxidative stress marker oxidative DNA adduct 8-hydroxy-2-deoxyguanosine (8-OHdG), both of which were associated with elevated levels of PM_2.5_ over a 24 h period. Notably, female participants demonstrated a more pronounced hs-CRP response to increased levels of PM_2.5_ compared to male participants.
Marshall Elizabeth G. [19]	690,000	Case-control study	O_3_ CO NO_2_ PM_10_ PM_2.5_	N.A.	The authors conducted a comparative analysis of estimated exposure to ambient air pollutants during early pregnancy between mothers of children with oral cleft defects (cases) and mothers of control subjects. They adjusted for available risk. The analysis revealed that higher quartiles of CO concentration demonstrated a consistent protective association with CPO (*p* < 0.01). For other contaminants, the 95% confidence intervals of the odds ratios for certain quartiles did not include one, indicating a potential association. CLP showed limited evidence of an association with increasing SO_2_ exposure, while CPO displayed weak associations with increasing O_3_ exposure.
Hwang Bing-Fang [20]	721,289	Population-based case-control study	SO_2_ NO_x_ O_3_ PM_10_	N.A.	Using geographic information systems, the authors employed the inverse distance weighting method to establish exposure parameters for SO_2_, NO_x_,O_3_, CO, and PM_10_ during the first three months of pregnancy. The authors expressed the effect estimates as odds ratios (ORs) per 10 ppb change for SO_2_, NO_x_, and O_3_, 100 ppb change for CO, and 10 μg/m^3^ change for PM_10_. The analysis revealed an increased risk of cleft lip and/or palate (CL/P) in relation to O_3_ levels during the first gestational month (adjusted OR = 1.20; 95% confidence interval, 1.02–1.39) and second gestational month (adjusted OR = 1.25; 95% CI, 1.03–1.52) within the range of 16.7 ppb to 45.1 ppb. However, there was no significant association observed with CO, NO_x_, SO_2_, or PM_10_.

**Table 4:** Table summarizing the principal air pollutants investigated.

Pollutant	Characteristics	Health Effects
PM_2.5_ and PM_10_	Particulate matter originating from primary emissions or secondary particles formed through physical and chemical reactions. PM_2.5_ and PM_10_ are extensively studied pollutants.	Heightens the risk of air pollution-related diseases, including acute respiratory tract infections, cardiovascular disease, chronic obstructive pulmonary disease (COPD), and lung cancer [21,22,23].
O_3_	Formed through complex photochemical reactions in the troposphere, particularly in the presence of pollutants and sunlight.	Acute effects include throat dryness, pharyngitis, bronchitis, reduced pulmonary bactericidal capacity, eye irritation, and cardiovascular effects. Chronic effects include fibrosis, teratogenic effects, and impacts on the reproductive system [24,25,26].
CO	Generated through incomplete combustion, characterized by its colorless and odorless nature.	Forms carboxyhemoglobin, leading to hypoxia, ischemia, and cardiovascular diseases. Symptoms of carbon monoxide poisoning include headaches, dizziness, weakness, nausea, vomiting, and loss of consciousness [25,27,28,29,30,31,32].
NO_2_	Emitted from combustion, primarily in urban areas, by automobile engines and power plants.	Causes irritation to the respiratory system, exacerbating respiratory symptoms, and resulting in hospitalizations. Prolonged exposure increases the risk of asthma development and susceptibility to respiratory infections, leading to respiratory diseases, breathlessness, bronchospasm, and pulmonary edema [27,28].
SO_2_	Emitted from the combustion of fossil fuels and industrial activities.	Adversely affects the respiratory system, leading to bronchitis, bronchospasm, increased mucus production, and aggravation of existing respiratory conditions [32].

## Abbreviations

O_3_	Ozone
NO	Nitrogen Monoxide
NO_2_	Nitrogen Dioxide
SO_2_	Sulfur Dioxide
CO	Carbon Monoxide
PM_10,2.5_	particulate matter
N.A.	Not applicable
